# Enabling Metabolomics Based Biomarker Discovery Studies Using Molecular Phenotyping of Exosome-Like Vesicles

**DOI:** 10.1371/journal.pone.0151339

**Published:** 2016-03-14

**Authors:** Tatiana Altadill, Irene Campoy, Lucia Lanau, Kirandeep Gill, Marina Rigau, Antonio Gil-Moreno, Jaume Reventos, Stephen Byers, Eva Colas, Amrita K. Cheema

**Affiliations:** 1 Biomedical Research Group in Ginecology, Hospital Universitari Vall d’Hebron, Institut de Recerca (VHIR), Universitat Autònoma de Barcelona, Barcelona, Spain; 2 Departments of Oncology and Biochemistry, Molecular and Cellular Biology, Lombardi Comprehensive Cancer Center at Georgetown University Medical Center, Washington, D.C., United States of America; 3 Institut d’Investigació Biomedica de Bellvitge (IDIBELL), Barcelona, Spain; 4 Gynecological Department, Vall Hebron University Hospital, Universitat Autònoma de Barcelona, Barcelona, Spain; 5 Department of Pathology and Molecular Genetics/Oncologic Pathology Group, Hospital Universitari Arnau de Vilanova, Universitat de Lleida, IRBLleida, Lleida, Spain; Mayo Clinic, UNITED STATES

## Abstract

Identification of sensitive and specific biomarkers with clinical and translational utility will require smart experimental strategies that would augment expanding the breadth and depth of molecular measurements within the constraints of currently available technologies. Exosomes represent an information rich matrix to discern novel disease mechanisms that are thought to contribute to pathologies such as dementia and cancer. Although proteomics and transcriptomic studies have been reported using Exosomes-Like Vesicles (ELVs) from different sources, exosomal metabolome characterization and its modulation in health and disease remains to be elucidated. Here we describe methodologies for UPLC-ESI-MS based small molecule profiling of ELVs from human plasma and cell culture media. In this study, we present evidence that indeed ELVs carry a rich metabolome that could not only augment the discovery of low abundance biomarkers but may also help explain the molecular basis of disease progression. This approach could be easily translated to other studies seeking to develop predictive biomarkers that can subsequently be used with simplified targeted approaches.

## Introduction

Most mammalian cell types secrete three types of extracellular vesicles either constitutively or in a regulated manner: exosomes, that are 35–150 nm diameter vesicles; ectosomes (also called microvesicles), from 100 to 1000 nm; and apoptotic bodies, from 500 to 2000 nm. Exosomes are formed from intraluminal vesicles and are delivered from multivesicular bodies to the outside of the cell by fusion with the extracellular membrane (endolysosomal vesicles) [1–3]. Microvesicles and apoptotic bodies originate by budding and fission of the plasma membrane. Exosomes are found in different biofluids including plasma, urine, cerebrospinal fluid, and uterine aspirates and were first described in 1983 by Pan BT et al. and Harding C et al. [4–5]. Exosomes contain proteins, nucleic acids, lipids, RNAs and small RNAs and metabolites and it is thought that their principal function is to facilitate cell-to-cell communication under normal and diseased conditions [6, 7]. They are rich in cell surface molecules that facilitate their fusion with the receptor membrane and release their cargo in the cytoplasm [8] and are constantly released into circulation or proximal biofluids under normal and diseased conditions, affecting either proximal or distant cells. Since exosomes can be easily enriched from biofluids and provide a fingerprint of their cell of origin, there is a growing interest in using exosomes for the identification of novel and specific biomarkers with potential utility for diagnosis and prognosis of different cancer types [9].

Although there is a general consensus in the scientific community about the use of serial ultracentrifugation as the method of choice to isolate exosomes, there are still limitations to confirm the intra-luminal origin of the isolated vesicles (i.e. mainly lack of specific biomarkers). Thus, enriched vesicles having similar morphology and size and unknown biogenesis are defined as Exosome-Like Vesicles (ELVs).

Groundbreaking research over last decade has delineated biomarkers that can be used for early detection of cancer, dementia as well as those that can be used for monitoring response to therapy [10, 11]. However, identification of biomarkers with high sensitivity and specificity for a given disease type, still remains a major challenge in the field [12]. Biofluid molecular profiling based approaches have intrinsic limitations for detection of low abundant biomarkers which are obscured by the presence of high abundance molecules in the matrix. Exosomes, on the other hand, offer promise as an untapped biomarker resource; given that enrichment of the exosome fraction is likely to alleviate the dynamic range issue that is a common analytical problem across a broad range of biomarker identification and characterization studies. Moreover, the exosome cargo is protected from nuclease and protease activity by a lipid bilayer, resulting in increased stability of the sample [13].

Several studies have described biomarkers associated with cancer cell related ELVs [14–16]. However, most comparative exosomal profiling studies with a case-control study design have focused on transcriptomic and proteomic techniques. Given that the ELVs membranes have a rich lipid and metabolite content, characterizing ELVs metabolomes from different biofluids is likely to provide new information that could be used for identification sensitive and specific biomarkers that would also serve as a phenotypic readout since metabolites represent the end point of cellular processes. A recently published proteoglycan study of the serum exosomal fraction has shown the value of this matrix as novel biomarker source with potential clinical utility [17].

Metabolomics is an emerging “omics” field that enables the identification and quantitation of a wide variety of small molecules that are indicative of metabolic, nutritional and physiological status of the patient. This analytical tool allows for the analysis of a large number of samples in a high-throughput manner, and consequently, permits the understanding of current molecular response of a biological system to any perturbation in its microenvironment [18]. Furthermore, combining chromatography coupled to electrospray ionization mass spectrometry and multivariate statistical analysis enables the detection of differential abundance of metabolites between two conditions and adds value to clinical and translational studies focusing on cost-effective, high through put biomarker development. Metabolomic profiling of human biofluids using the enriched ELVs fraction represents a surrogate for tissue biopsy as a non-invasive tool for low abundance biomarker discovery and validation studies. However, there are few metabolic characterization studies reported in literature augmenting these investigations.

In this study, we have characterized ELVs metabolomes derived from human plasma samples which is a widely used matrix for biomarker studies. In addition, we also analyzed the metabolome of ELVs isolated from cell culture media since cell lines are widely used as a model system in biomedical research. Moreover, we report the differences on the ELVs metabolomics profile when comparing two conditions: endometrial cancer (EC) patient plasma samples versus control subjects; TGF-β treated pancreatic cancer human cell lines versus matched controls as two proof of principle studies. To our knowledge, this is the first report describing the presence and composition of metabolite cargo of ELVs derived from plasma and cell culture media using a high resolution mass spectrometry approach. This method development effort represents a first yet critical step for biomarker discovery using exosomal metabolomics with wide applicability. In addition to performing extensive characterization of metabolomic content of ELVs in plasma, we also present an approach that can be generically used for biomarker discovery and validation studies.

## Materials and Methods

### Patients

Participants in the study attended the Department of Gynecologic Oncology at the Hospital Vall Hebron in Barcelona, Spain. None of the patients included in the study received treatment prior to the collection of the biofluids. Final diagnosis was performed in the Department of Pathology at the same hospital. All patients participating in the study signed an informed consent. The Clinical Research ethics Committee at the Hospital Universitari Vall d’Hebron (Barcelona, Spain) approved the study. Collection of biofluids for the study included 10 mL of blood from post-menopausal cancer patients, with endometrioid adenocarcinoma, and control subjects. Samples were drawn from patients under sterile conditions, de-identified for research purposes and were processed, aliquoted and frozen at -80°C within four hours of collection. A full description of the clinic pathologic features of the patients included in both studies are detailed in S1 Table.

### Plasma collection

Blood samples were collected in 10 mL EDTA-tubes (Cat# 367525. Pulmolab, CA, USA) and inverted 10 times at room temperature. Protease inhibitors (1:200, Cat# P8340. Sigma Aldrich, MO, USA.) were added to the sample. The sample was then centrifuged at 1,300 g during 15 min at room temperature. The supernatant was transferred to a clean tube and centrifuged at 3,000 g for 15 min at 4°C. The supernatant (plasma) was aliquoted and frozen at -80°C.

### Cell culture

Human pancreatic carcinoma epithelial-like cell line PANC1 (ATCC^®^ CRL-1469™) was obtained from the Tissue Culture Shared Resource of Georgetown University (Washington DC, USA). PANC1 cells were grown in DMEM media supplemented with 10% fetal bovine serum (HyClone, Logan, UT, USA) and penicillin/streptomycin. Reagents were obtained from HyClone (Logan, UT, USA). Cells were grown in a 5% CO2 incubator at 37°C.

Cells treatment: Cell lines were grown to 60% confluence, serum starved for 24 h and treated with 10 ng/mL of TGF-β (Cat # P01137, R&D Systems, MN, USA) for 48 h along with matched controls. Cells then were harvested and collected in 1.5 mL tubes. Samples were centrifuged at 13,000 rpm for 5 min, supernatant was removed, and cells were kept at -80°C.

### Real-Time Reverse Transcription PCR

RNA was purified using the detailed protocol of RNeasy Mini Kit (Cat # 74104, Qiagen, CA, USA) and quantified by NanoDrop (NanoDrop 2000c, Thermo Scientific, NY, USA). Reverse-Transcription Reaction was performed following the standard protocol of RT2 First Strand Kit (Cat # 330401, Qiagen, CA, USA). The cDNA obtained was analyzed by Real Time PCR (Cat # 4309155, SYBR Green, Applied Biosystems, CA, USA). Primers used for the Real Time PCR: GAPDH-F 3acggatttggtcgtattggg5, GAPDH-R 3tgattttggagggatctcgc5, E-cadherin-F 3tgcccagaaaatgaaaaagg5, E-cadherin-R 3gtgtatgtggcaatgcgttc5, N-cadherine-F 3ggacagttcctgagggatca5, N-cadherine-R 3ggattgccttccatgtctgt5, Vimentin-F 3ggctcagattcaggaacagc5, Vimentin-R 3gcttcaacggcaaagttctc5.

### ELVs isolation

For ELVs isolation from cell culture, 30 mL of media was centrifuged (for three biological replicates), for 5 min at 300 g in order to remove the cell debris followed by another centrifugation at 2500 g for 20 min. ELVs isolation was performed for cell media and plasma using a modification of the protocol published by Thery et al [19]. Briefly, samples were centrifuged at 16,500 g at 4°C for 20 min to remove cell debris and fine particulates; subsequently the supernatant was placed in an ultracentrifuge tube (Cat#0314, Thermo Scientific, NY, USA) and centrifuged at 100,000 g at 4°C for 2 h. The pellet obtained was washed carefully with Tris Buffered Saline (TBS)-Ca2+ and centrifuged again at 100,000 g at 4°C for 1 h. Finally ELVs (pellet) were re-suspended in 60 μL PBS 1X buffer and frozen at -80°C. Although, exosome isolation can be performed using commercially available kits; many of these use Polyethylene Glycol (PEG) which interferes with downstream mass spectrometry based analysis.

### Nanoparticle Tracking Analysis

The size and number of ELVs was determined using a Nanosight LM10 instrument with Nanoparticle Tracking Analysis (NTA, Malvern Instruments, UK). ELVs were diluted with Milli-Q water (Milli-Q Synthesis, Merck Millipore, Massachusetts, USA) and the analysis was performed following manufacturer’s instructions. The videos were recorded for 1 min. Measurements were repeated at least 3 times per sample. The data were analyzed using the version 2.3 of the NTA-software. The size of the particles was calculated automatically based on the Brownian motion rate.

### Western blot

Protein extraction of total plasma and plasma ELV fraction was performed by adding RIPA buffer, incubating at 4°C for 1 h followed by sonication. The supernatant containing exosomal proteins was collected after 15 min of centrifugation at 15,000 rpm. Protein concentration was determined using the standard protocol for the Bradford colorimetric method. Samples were loaded onto SDS-PAGE gels and transferred to a PVDF membrane. Membranes were blocked with 5% non-fat dried milk in TBS-Tween (0.01%) for 45 min and incubated overnight at 4°C with the primary antibodies, washed with TBS1x, incubated at room temperature with the secondary antibody and revealed.

Reagents. Primary antibodies: Flotillin-1 (1:250, Cat# 610821, BD Biosciences, San Jose, CA, USA), TSG101 (1:500, Cat#ab83. Abcam, MA, USA), CD63 (1:1000, Cat#OP171. Calbiochem, Merck Millipore, Massachusetts, USA), CD9 (1:250, Cat#555370. BD Biosciences, San Jose, CA, USA), Rab5 (1:2000, Cat#ab13253, Abcam, MA, USA), CD81 (1:1000, Cat#sc-166028. Santa Cruz, Texas, USA) and Haptoglobin (1:1000, Cat#ab131236, Abcam, MA, USA). Secondary antibodies: Goat anti-rabbit, Cat# P0448; rabbit anti-mouse, Cat# P0260, both 1:2000. Dako, CA, USA). RIPA buffer (5nM EDTA, 150mM NaCl, 1% Triton, 20nM Tris pH8 and 1:200 protein inhibitors). Immobilon Western Chemiluminiscent (Cat#WBKLS0100. Merck Millipore, Massachusetts, USA).

### Metabolomic analyses of ELVs and database search

ELVs isolated from plasma and cell culture media were processed for mass spectrometric analysis as described by Sheikh *et al* with minor modifications to the original protocol [20]. Initially, samples were thawed on ice and vortexed. For metabolite extraction, 55 μL (v/v) of ELVs containing approximately 10^11–12^particles/mL were mixed with 95 μL of water. The tubes were placed on dry ice for 30 sec followed by 90 sec incubation in a 37°C water bath. The samples were sonicated for 30 sec. A total of 600 μL of chilled methanol containing internal standards (5 μL of debrisoquine at a concentration of 1mg/mL and 25 μL of 4-nitrobenzoic acid at a concentration of 1mg/mL) were added to each sample. The coefficient of variation (% CV) of each internal standard for each matrix and condition was calculated (S2 Table), which ranged between 5.5 to 13.83%. Following vortexing, samples were incubated on ice for 15 min and 600 μL of chloroform were added to each tube, followed by centrifugation at 13,000 rpm at 4°C for 10 min. The supernatant was transferred to a fresh tube and 600 μL of chilled ACN were added. Samples were incubated at -20°C overnight and centrifuged at 13,000 rpm at 4°C for 10 min. The supernatant was transferred to a fresh tube and dried under vacuum. This sequential extraction strategy [going from water (aqueous) to methanol (semi-polar) to non-polar solvent extraction (chloroform)] allows for a broad range extraction of metabolites. Dried samples were resuspended in 200 μL of 50% water and 50% methanol followed by UPLC-ESI-Q-TOF-MS analysis. The solvent composition supports the dissolution and MS analysis of a broad class of compounds. A total of 5 μL of each sample were injected onto a reverse phase Acquity UPLC CSH C18 1.7 μm, 2.1 x 100 mm column (Waters Corp.). Two different gradients were used based on the sample matrix (plasma and cell culture media). MS data acquisition was performed using ESI-QTOF MS within the mass range of 50 to 1200 mass-to-charge ratio (m/z) in positive and negative electrospray ionization modes on a (Xevo G2 Q-TOF; Waters Corp.). MS data were pre-processed using the XCMS software [21]. The Madison Metabolomics Consortium Database (MMCD) [22], the Human Metabolome Database (HMDB) [23], LIPID MAPS [24] and Metlin [25] were used for accurate mass based putative identification of metabolites. We performed a multivariate data analysis using Metaboanalyst 3.0 web tool [26]. Identities for a sub-set of metabolites were validated by tandem mass spectrometry.

Reagents for MS analysis: Chloroform, LC/MS-grade acetonitrile (ACN), water and methanol were purchased from Fisher Optima grade, Fisher Scientific (New Jersey, USA). PBS was purchased from Invitrogen (Carlsbad, CA, USA). High purity formic acid (99%) was purchased from Thermo-Scientific (Rockford, IL, USA). Ammonium formate, debrisoquine and 4-Nitrobenzoic acid (4-NBA) were purchased from Sigma- Aldrich (St. Louis, MO, USA).

## Results

### Characterization of plasma ELVs

**Fig 1:**
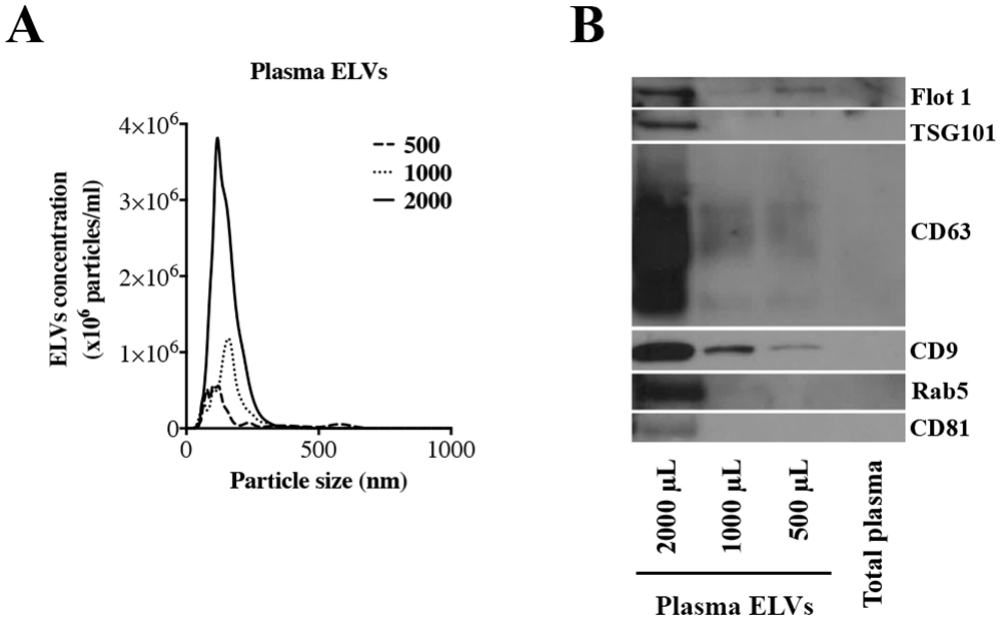
Nanoparticle Tracking Analysis (NTA). Determination of the concentration and particle size of human from 500, 1000 and 2000 μL of human plasma **(Panel A)**. Size distribution of the nanovesicles was around 150 nm. **Western blot analysis to confirm the expression of exosome specific markers (Panel B)**. Human plasma ELVs enrichment confirmed in exosomes isolated from different volumes of human plasma (2000, 1000 and 500 μL) and no presence of exosomal specific markers when loading total plasma (non-enriched). Lane A through C represent ELVs isolated from 2000, 1000 and 500 μL of plasma respectively; Lane D represents non-enriched total plasma.

Procuring human plasma samples from biorepositories for biomarker discovery efforts is often constrained by sample volumes and associated costs of the study; hence our initial goal was to optimize minimum volume of plasma for ELVs isolation suitable for downstream metabolomic analysis using high resolution mass spectrometry. For this purpose, we isolated ELVs from different volumes of human plasma (500, 1000 and 2000 μL) as described in methods section [19]. For all samples, we determined size distribution and concentration of the ELVs enriched fraction by NTA, protein concentration and total protein amount. The average size of plasma ELVs was approximately 150 nm (Fig 1A). The final concentration of ELVs correlated with the starting volume, ranging from 1x10^12^ particles per mL for 500 μL samples to 7.5x10^12^ particles per mL for 2000 μL of plasma. These results are consistent with previous studies that have reported similar size and number of particles per mL of plasma [27, 28]. Equal volumes of protein extract corresponding to ELVs purified from 2000, 1000 and 500 μL of plasma were subjected to SDS-PAGE. The gel was also loaded with protein extracted directly from total plasma (Fig 1B) in order to show the enrichment of exosome specific protein markers in the purified samples. For the total plasma we loaded the same amount of protein that we loaded for the protein extract corresponding to ELVs purified from the higher volume (2000 μL). The non-enriched plasma sample does not show the presence of ELVs specific markers while the enriched fractions exhibit the presence of these markers in a concentration dependent manner which in turn was dependent on the initial volume of plasma that was used for enrichment of the ELVs fraction. These include Flotillin 1, TSG101, CD63, CD9, Rab5 and CD81 (Fig 1B). However, as seen in the figure, the concentration of ELVs in non-purified plasma samples is too low for detection of these markers by immunoblotting. Uncropped western blots are shown in S1A and S1B Fig. In order to understand the amount of co-pelleted artifacts at each step of the isolation method, we performed an immunoblot against haptoglobin (S1C Fig), a soluble protein found in plasma, with MVs and ELVs isolated from 2000, 1000 and 500 μL of plasma. As we expected, we detected the presence of haptoglobin in MVs, since they are collected after the first round of centrifugation (16,500 g for 20 min) where we get rid of the large vesicles and molecules contained in the sample. However, the following steps of ultracentrifugation permitted to rule out most of the contaminants. We could also detect the presence of haptoglobin in ELVs isolated from large volumes of plasma (2000 μL), but not from 1000 and 500 μL. These results strongly suggest the enrichment of ELVs from plasma that were ready for metabolomics characterization using high resolution mass spectrometry.

**Fig 2:**
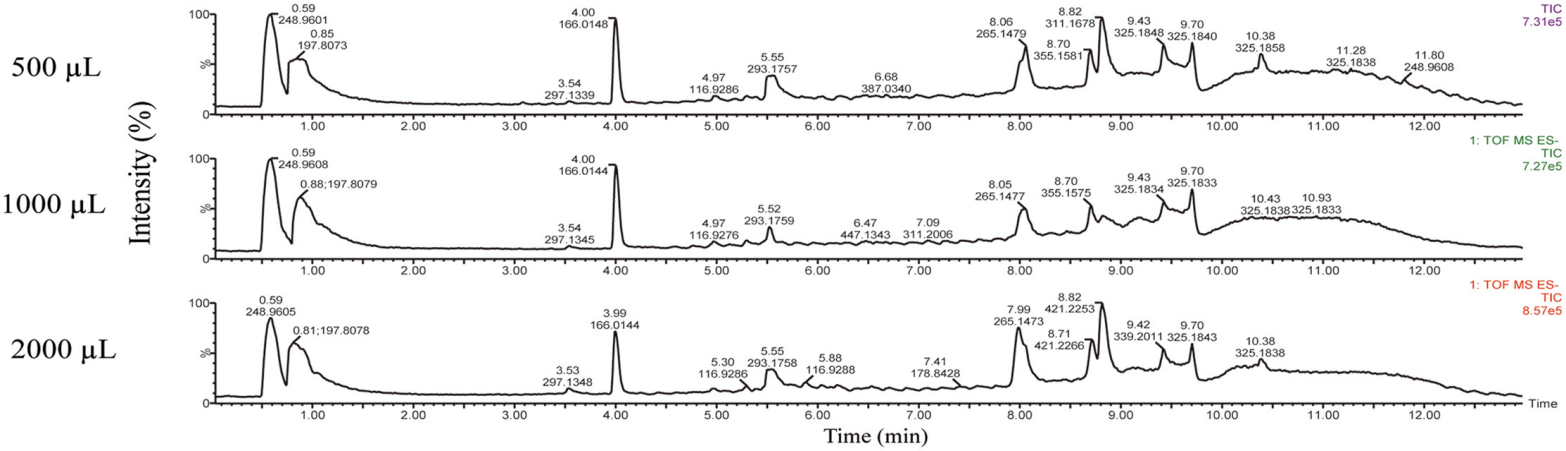
Total Ion Chromatograms (TIC) of human plasma Exosome-Like Vesicles (EVLs) in the negative electrospray ionization mode. ELVs were isolated from different volumes of human plasma (500, 1000 and 2000 μL) and subjected to TOF-MS analysis. The X-axis represents the chromatographic retention time while the Y-axis represents the relative intensity.

The Total Ion Chromatograms (TICs) observed in the negative ionization mode for the metabolites extracted from the ELVs of 500, 1000 and 2000 μL of plasma are shown in Fig 2. The ratio of relative intensity values for some features detected are shown in S3 Table. Although with 1 or 2 mL of plasma, a small fraction of metabolites showed improved ion intensity, a large number of detected metabolites did not show any changes in relative ratios or improvement signal to noise ratio for individual metabolites suggesting that 500 μL of plasma was optimal for performing metabolomics profiling studies using matrices of human plasma samples.

**Fig 3:**
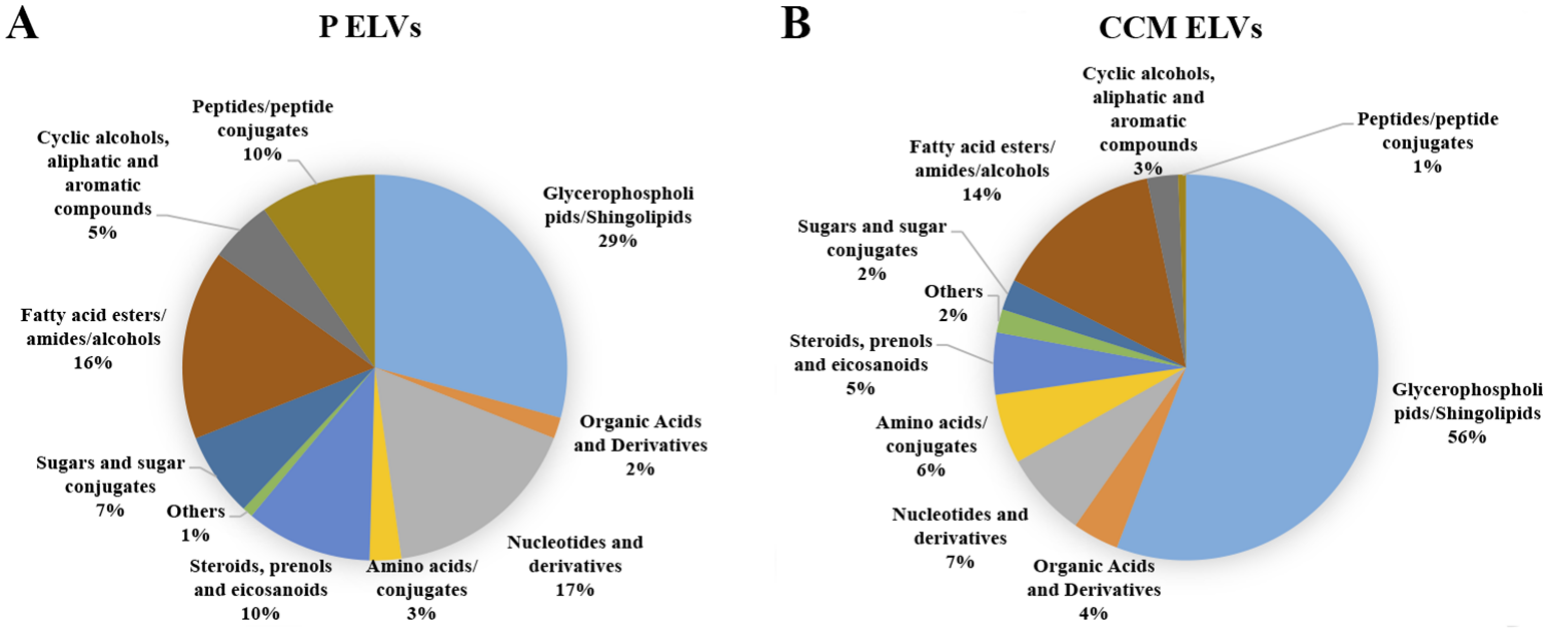
Metabolomic ELVs composition. Characterization of the metabolite content of human plasma Exosome-Like Vesicles (P ELVs) **(Panel A)** or PANC 1 cell culture media (CCM ELVs) **(Panel B)**.

Hence further metabolomics characterization was performed with ELVs isolated from 500 μL of plasma samples that were analyzed using quadrupole time of flight mass spectrometry using three biological replicates. The total number of features detected in plasma derived ELVs samples was 840 and 2198 in the negative and positive ionization mode, respectively. Next, we annotated the features by performing an accurate mass based database search and an enrichment analysis using Metlin and Human Metabolome Databases (S4A and S5 Tables). Our results showed that the metabolome of ELVs derived from plasma maintained a high prevalence of glycerophospholipids (possibly representing exosomal membrane contents) and shingolipids, representing 29% of the total metabolome composition, such as PI (16:0/22:4), PE (22:2/16:1), GalCer (d18:2/16:0), GPCho (18:0/14:0) or TG (12:0/12:0/20:5). The fatty acid esters, amides and alcohols (oleamide, malonyl-CoA among others) represented 16% of the total metabolites detected in this analytical run while the nucleotides, nucleosides and their derivatives comprised 17% of the metabolome. Peptides, organic acids and derivatives were less abundant in the total ELVs metabolite content (Fig 3A). Some of the interesting metabolites identified in plasma ELVs included coenzyme Q10, ubiquinone 9, palmitoyl glucuronide, PG (16:0/16:0), 25-hydroxy-hexadehydrovitaminD3, 10-formyldihydrofolate, acetyl glucosamine bisphosphate, malonyl-CoA, cytidine-5'-monophosphate, N-Arachidonoyl-L-Serine, tyrosyl-AMP, oleamide, deoxyuridine-diphosphate, picolinic acid and deoxyvitamin D3 and SM (d18:1/16:0) indicating a wide class of metabolites. A subset of these metabolites was validated using tandem mass spectrometry (Table 1). Some of these metabolites fall below the detection limits in routine untargeted metabolomics profiling of plasma samples, possibly because of ion suppression in presence of other high abundant metabolites that get eluted in the same chromatographic time scale.

**Table 1:** Validated metabolites.

Metabolite	Metabolite ID	m/z	Mass error (ppm)	Retention time (min)	Matrix	Mode	Major CID fragments
PG(16:0/16:0)	123064911 (Pubchem SID)	707.520	0.002	10.129	P ELVs	Pos.	663.4540, 607.3931, 551.3286, 495.2679, 439.2032
N-Arachidonoyl-L-Serine	74380323 (Pubchem SID)	392.280	0.001	9.252	P ELVs	Pos.	149.0277, 123.0987, 106.0971, 95.0809, 69.0711, 57.0866
SM(d18:1/16:0)	7850646 (Pubchem SID)	703.574	0.001	9.993	P ELVs	Pos.	184.074
Oleamide	C19670	282.279	0.000	8.020	P and CCM ELVs	Pos.	97.1005, 83.0866
Coenzyme Q10	C11378	861.677	0.000	10.301	P ELVs	Neg.	724.8752, 588.8987, 520.9148, 452.9229, 384.9373, 316.9473, 248.9610, 180.9727, 112.9871
Malonyl-CoA	cq_00054	853.593	0.001	10.026	P ELVs	Neg.	808.727
All-trans-4-oxoretinoic acid	HMDB06285	315.195	0.001	4.327	CCM ELVs	Pos.	203.1072, 187.1123
Psychosine sulfate	5280538 (Pubchem SID)	542.301	0.001	4.049	CCM ELVs	Pos.	427.2240, 358.1841, 255.1232, 249.1212, 155.1072, 123.0446

List of metabolites contained in plasma Exosome-Like Vesicles (P ELVS) and PANC 1 cell culture media Exosome-Like Vesicles (CCM ELVs) confirmed by MS/MS.

### Characterization of ELVs from cell culture media

In order to characterize ELVs in an *in vitro* model system, we isolated ELVs from cell culture media as described in the methods section. The ELVs were characterized using NTA, western blotting and finally subjected to high resolution mass spectrometry based analysis as described for plasma ELVs.

**Fig 4:**
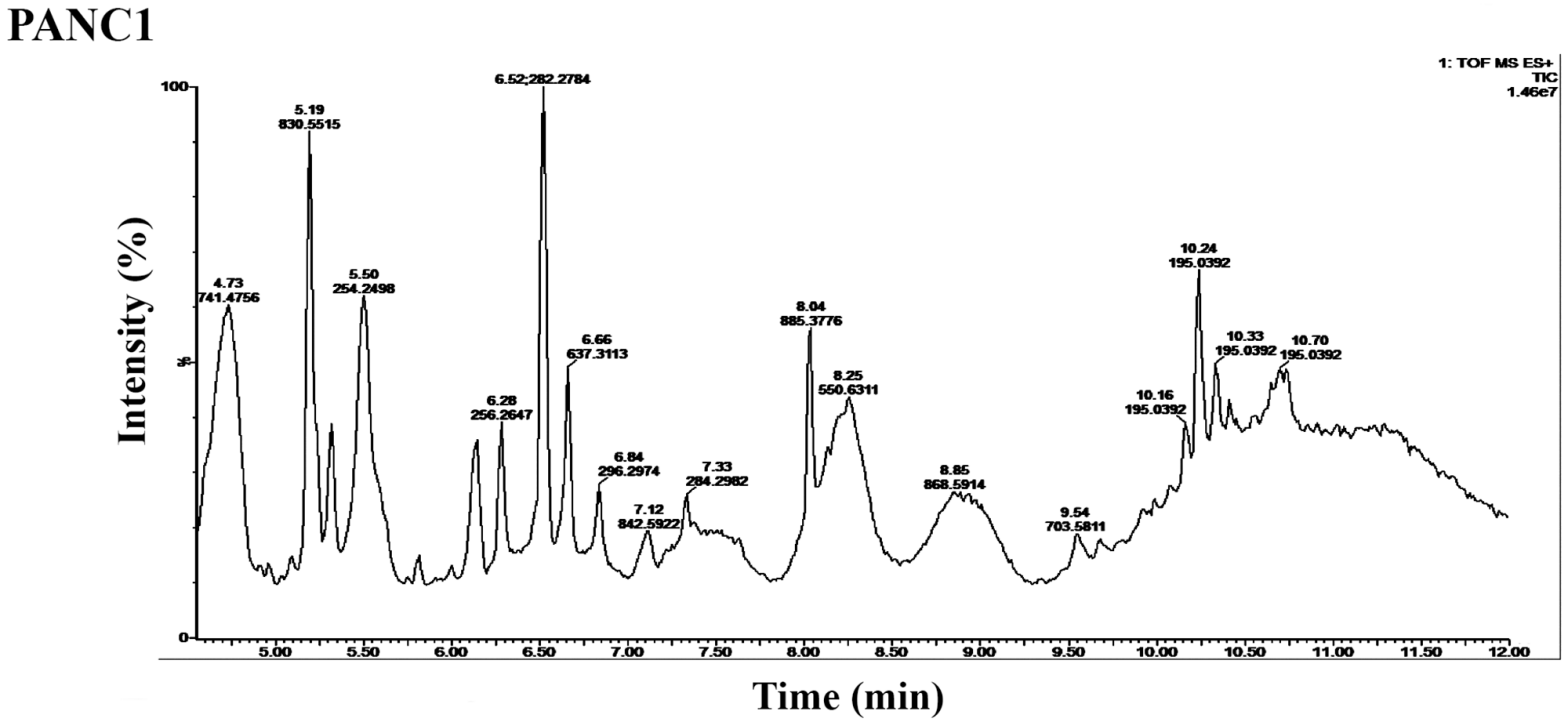
Total Ion Chromatograms (TIC) of PANC1 pancreatic cancer cell culture media Exosome-Like Vesicles (ELVs) in the positive electrospray ionization mode. ELVs were isolated from pancreatic cell line PANC 1 media and subjected to TOF-MS analysis. The X-axis represents the chromatographic retention time while the Y-axis represents the relative intensity.

We analyzed the bio-composition of ELVs that were isolated from cell culture media obtained by growing PANC1, a human pancreatic cell line as described in the methods section. We used three biological replicates for these analyses. The ELVs for each sample were isolated from 30 mL of media. Quantitative analysis of the exosomes isolated using NTA revealed an average size of 95 nm and average concentration of 1.6 x10^10^ particles per mL consistent with the size of ELVs previously reported. The Total Ion Chromatogram (TIC) observed in the positive ionization mode for PANC1 is shown in Fig 4. Data were pre-processed using XCMS software that yielded 596 and 2926 features in the negative and positive electrospray modes respectively.

Database search of ELVs metabolome obtained from cell culture media was performed (S4B and S5 Tables) and dominated by a majority of glycerophospholipids and shingolipids (56%) and a significant representation of fatty acid esters, amides and alcohols (14%) and nucleotides and derivatives (7%). We also found a representation of sugars, cyclic alcohols, aromatic compounds, steroids and organic acids which are generally suppressed when whole cell extracts are used for molecular profiling (Fig 3B). In addition we were able to detect Adenine, aminoadipic acid, enol-Phenylpyruvate, oleamide, 15-HETrE, 3-Dehydrosphinganine, all-trans-4-oxoretinoic acid, N,N-dimethylsphingosine, docosanamide, psychosine sulfate, pentaglutamyl folate and fructose 1,6-bisphosphate. Some of these metabolites were unambiguously identified by comparing fragmentation patterns of metabolites acquired in the elevated collision energy mode (MS^E^) using mass fragment calculator (Table 1). These results emphasize the presence of a broad class of metabolites with potential to be used as biomarkers.

### ELVs metabolome as a biomarker source

Having characterized the metabolome of ELVs isolated from plasma and cell culture media, our next goal was to test the possibility of using comparative ELV metabolomic/lipidomic profiling to distinguish between two or more groups. We used two proof of principle studies; the first study was designed to delineate differences in the ELV profiles enriched from PANC1 cells treated with transforming growth factor beta (TGF-β) to those that were untreated while in the other we compared the metabolomic profiles of ELVs isolated from plasma of EC patients to those obtained from healthy volunteers.

#### Comparative Profiling of TGF-β treated PANC1 cells

**Fig 5:**
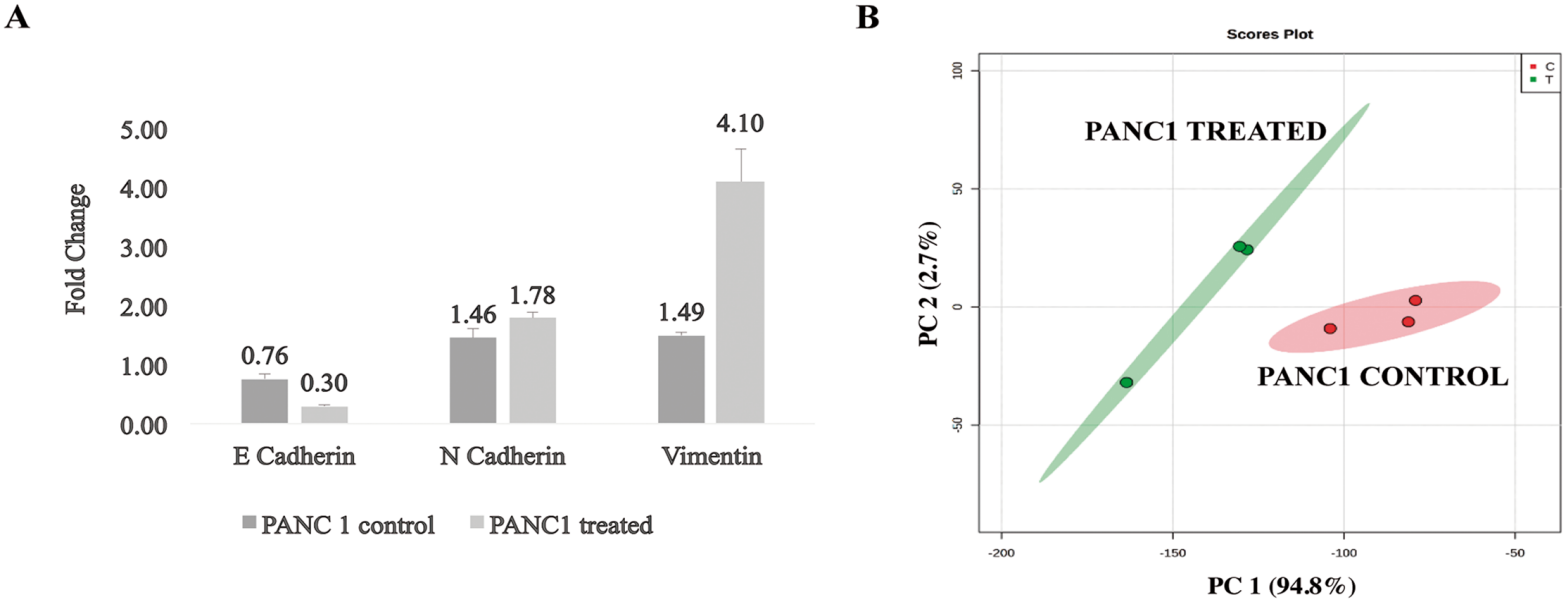
**Panel A. TGF-beta induces epithelial to mesenchymal transition (EMT) in PANC1 cells**. Real Time PCR analysis showed a decrease of the epithelial marker E-cadherin (p-v<0.05) and an increase of the mesenchymal markers N-Cadherin and Vimentin (p-v<0.05) expression after TGF-β treatment in PANC1 cells. **Panel B**. **Principal Component Analysis (PCA) of exosomal metabolome for MS negative ionization mode showing the separation between the two study groups**. TGF-β treated (T) and control (C) PANC1 cells. We analyzed three replicates per condition. The x-axis shows interclass separation while y-axis illustrates the intra-class variability on Y-axis.

It has been demonstrated that epithelial to mesenchymal transition (EMT) contributes to metastasis and treatment resistance of pancreatic cancer cells [29]. It is also well known that TGF-β acts as a driver in cancer progression through induction of EMT [30]. Therefore, we treated PANC1 cells with TGF-β, while the control cells were treated with DMSO and interrogated the metabolomic alterations in ELVs isolated from spent media under these two experimental conditions. We used three biological replicates for each condition (treated and untreated). The cells and media were collected after treatment (see methods). The cells were tested for EMT induction by TGF-β while the media were used for ELVs enrichment for subsequent metabolomic analyses. As expected, at RNA level we observed downregulation of the epithelial marker E-cadherin and the overexpression of the mesenchymal marker Vimentin in TGF-β treated cells compared to the controls (Fig 5A). Next we isolated ELVs from cell culture media and using an untargeted metabolomic approach we were able to elucidate a variation of the ELVs metabolome in response to TBF-β treatment. The principal component analysis (PCA) obtained shows a clear separation of the metabolomic composition of the ELVs due to the treatment (Fig 5B). Metabolites showing significant differences (fold change (FC) ≥ 2 and p-value ≤ 0.05) are listed in S6 Table. UDP-D-glucosamine (m/z: 550,084) and DG (20:2)/18:1/0:0) (m/z: 647,544) were two of the metabolites identified to be significantly up-regulated after TGF-β treatment.

#### Metabolomics of plasma ELVs reveals differences between controls and EC patients

**Fig 6:**
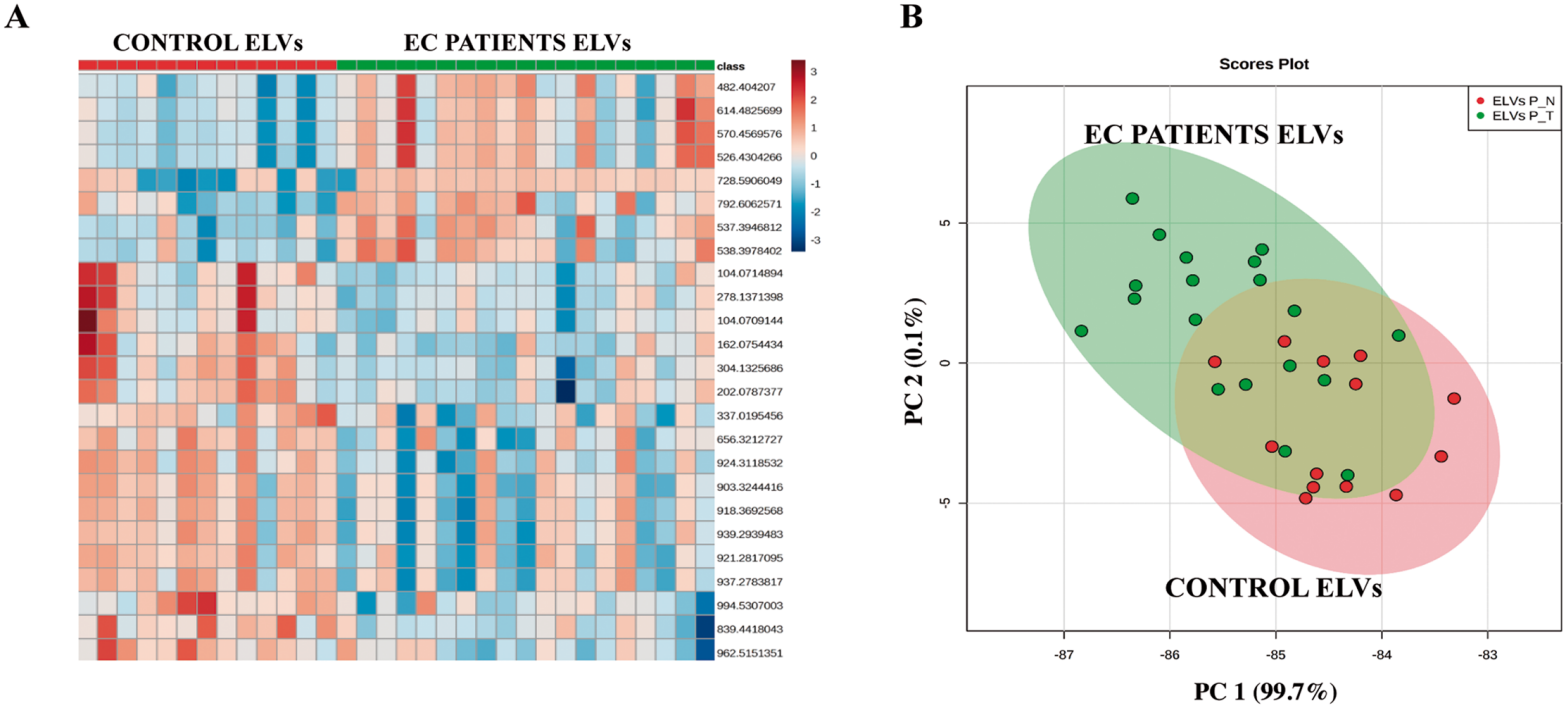
Multivariate analysis reveals distinct metabolic changes in plasma derived Exosome-Like Vesicles (ELVs) isolated from endometrial cancer (EC) patients compared to the control subjects. **Panel A**. Heat map visualization of ion rankings of volcano plot based m/z, corresponding to their relative levels (intensity) in plasma ELVs isolated from EC patients and control subjects for MS positive ionization mode. Each row on the heat map represents a unique feature with a characteristic mass to charge ratio and retention time while each column represents one subject. **Panel B**. Principal Component Analysis (PCA) plot for MS positive ionization mode showing the separation between EC and control plasma ELVs. The x-axis shows interclass separation while y-axis illustrates the intra-class variability.

We hypothesized that a compendium of molecular profiles (metabolomics) would provide insight into alterations that underscore the tumor phenotype in EC patients. Therefore, as a proof of principle, we compared metabolomic profiles of ELVs isolated from plasma of 13 control subjects (healthy patients) and 19 EC patients that were analyzed by UPLC-ESI-Q-TOF-MS. Multivariate analysis was performed in order to elucidate the differences in the ELVs metabolome composition in the two study groups. The differential abundance of metabolites was visualized as a heat map (Fig 6A) and PCA (Fig 6B) which indicated a differential pattern of the metabolites found in the ELVs isolated from EC plasma patients compared to the controls. Several significant metabolites did not yield an accurate mass based putative identification when searched against several databases which remains a major challenge in the field. The differentially abundant metabolites with putative IDs included substituted sugars and amino acids. Further characterization and validation of these findings is ongoing in our laboratory.

## Discussion

In this study, we present a broadly applicable approach for metabolomic profiling of ELVs isolated from human plasma as well as cell culture media. We chose these two matrices for optimizing metabolomics methodologies since these are widely used in studies with clinical and basic science focus respectively. Furthermore, the use of ELVs as a biomarker resource is useful since they can be isolated from most bodily fluids and their rich content is protected from degradation, allowing for circulation as well as for cell-to-cell communication of molecules and metabolites that would not be stable in free plasma and other biofluids [31]. It has also been reported that the ELVs secretion rate derived from tumoral cells is much higher than the rate for healthy cells; being involved in signal transmission in tumoral microenvironment [32].

We started out by confirming isolation and enrichment of ELVs fraction from human plasma and cell culture media by immunoblotting. Next, we characterized their size and concentration by Nanoparticle Tracking Analysis. A set of common and well established ELVs markers were identified in the extractions enriched in vesicles around 95–150 nm confirming the specific enrichment of ELVs. We also determined that 500 μL volume of plasma is optimal for generating high quality MS data for profiling the metabolomic and lipidomic content of the ELVs; increasing plasma volumes further does not add significant value to the number and signal to noise ratio of the detected metabolites.

A sub-set of the metabolites identified in plasma ELVs were validated including PG (16:0/16:0), N-arachidonoyl-L-serine, SM (d18:1/16:0), coenzyme Q10 and malonyl CoA. PG (16:0/16:0) is a glycerophospholipd precursor of cardiolipin. Cardiolipin is found in the inner mitochondrial membrane and a change in its concentration and distribution in this organelle is known to cause several diseases including cancer and aging [33]. It has also been reported that the endocannabinoid N-arachidonoyl-L-serine can have a neuroprotective effect in maintaining the undifferentiated state of some cells [34] and can promote cell proliferation, migration and angiogenensis [35]. Moreover, recent studies have revealed that Coenzyme Q10, apart from its antioxidant functions, also regulates the expression of genes involved in cell signaling and transport. The incorporation of Coenzyme Q10 in experimental lipovesicles enhanced their cell uptake [36] and an increase of Coenzyme Q10 concentration in vesicles prolonged their circulation in blood [37].

Furthermore, we analyzed the biochemical composition of cell culture media ELVs and we were able to identify some metabolites such aminoadipic acid that is an intermediate metabolite in the lysine pathway. It acts also as an antagonist of the neuroexcitatory activity and inhibits the production of kynurenic acid in some tissues [38]. Enol-phenylpyruvate was also present in cell culture ELVs and is related to phenylalanine and tyrosine metabolism pathways as well as to macrophage induced inflammatory processes [39]. We found also 15-HETrE, which is a polyunsaturated fatty acid involved in tumorigenesis and in the modulation of arachidonic acid metabolism. It also regulates the activity of cyclooxygenase-2 by inhibiting its expression [40]. Finally, all-trans-4-oxoretinoic acid was also present in the ELVs. It is an isomer of retinoic acid that is actively involved in cell-to-cell communication by modulating gap junctional activity [41]. There was an overlap in the different classes of metabolites obtained from plasma and cell culture media ELVs although the distribution across different classes was matrix dependent (Fig 3).

**Fig 7:**
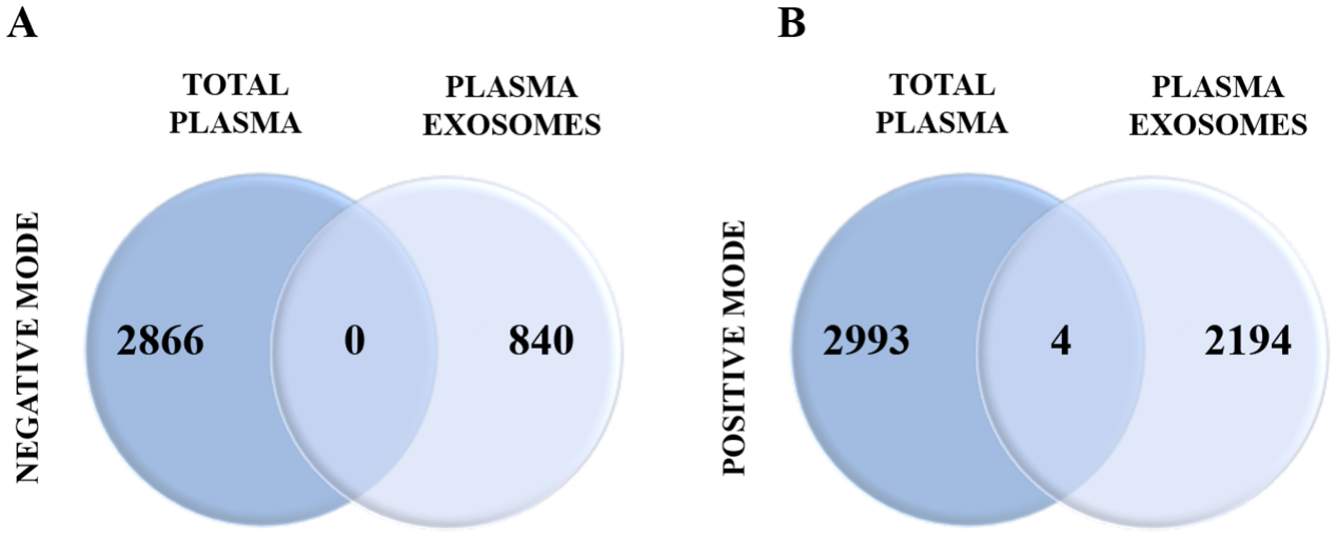
Comparison of the number of features detected in total plasma (non-purified) or plasma ELVs. The number of common and unique features for each type of sample is represented for the negative (Panel A) and positive (Panel B) electrospray ionization mode.

**Fig 8:**
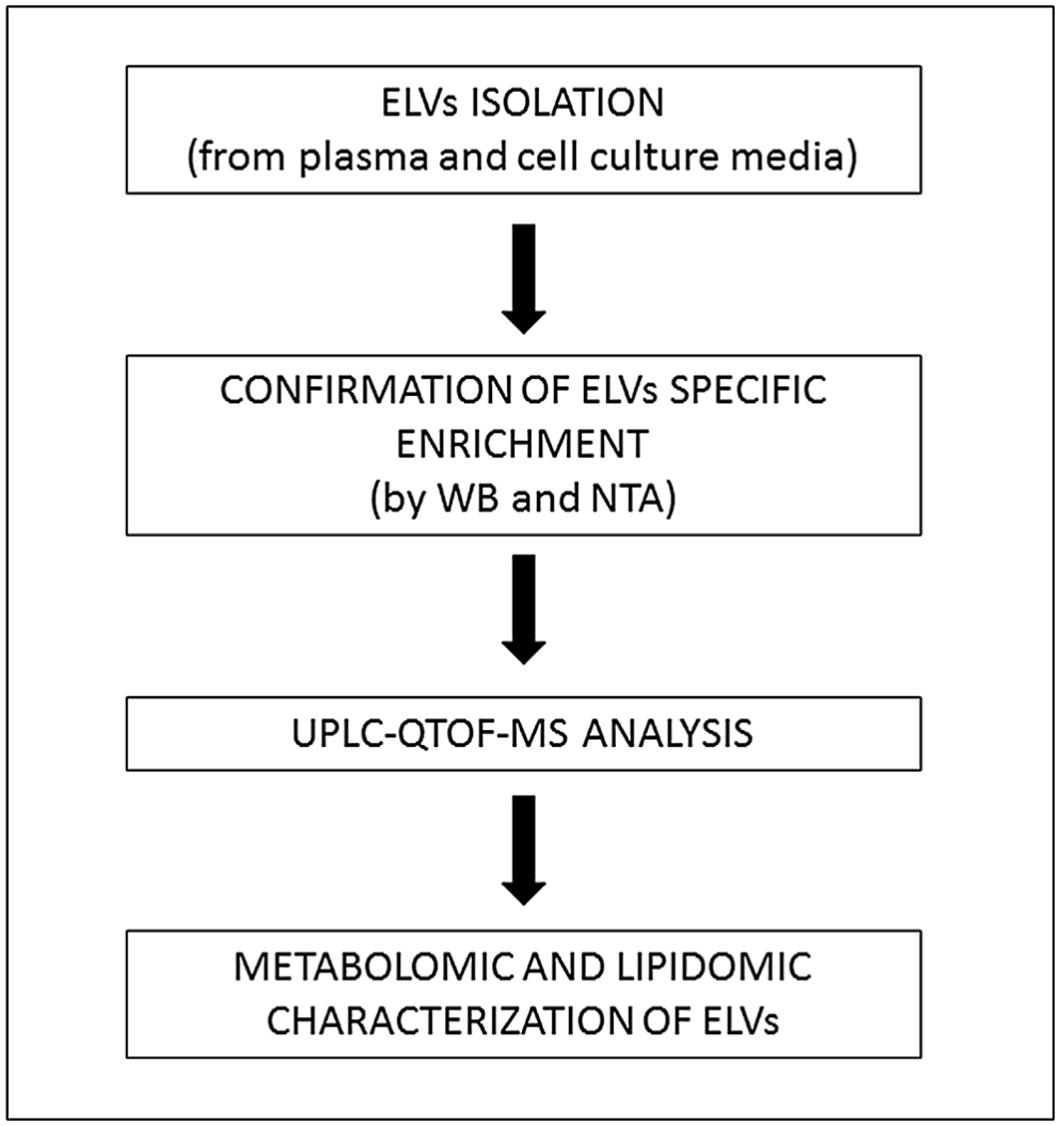
Workflow. Schematic showing experimental design in order to analyze the composition of exosome-like vesicles (ELVs) derived from plasma samples and cell culture media. (WB = western blot, NTA = nanoparticle tracking analysis).

As expected, the major class of metabolites were glycerophospholipids, which are integral components of the exosomal membranes and could majorly contribute to high phospholipid content associated with human plasma samples. Interestingly, we also found metabolites that belong to different classes including organic acids (several glycolytic intermediates), cyclic alcohols, steroids, prenols and amino acid conjugates as well as sugar and sugar conjugates (S4A and S4B Table) both in plasma and cell culture media ELVs. In our experience with routine plasma profiling experiments many of these metabolites fall below the instrumental limit of detection. Hence we compared the number of features obtained from regular plasma profiling with those obtained from ELVs fraction enriched from 500 μl of plasma (Fig 7). There was little overlap of detected metabolites between the two matrices most probably because of sensitivity range of the instrument for detecting metabolites from 25 μl of human plasma that we routinely use for untargeted metabolomics profiling [42]. This underscores the importance of enriching this fraction for low abundance biomarker discovery using plasma samples. Thus, detection of these compounds with significant implications in cell-to-cell communication and signaling, regulation and metabolic status is of critical importance for novel, low abundance biomarker discovery. We further propose that these compounds can be further characterized from whole plasma extracts using multiple reaction monitoring based targeted mass spectrometry thus dramatically increasing the sensitivity and specificity of the assay as well as the flexibility of multiplexing such that multiple metabolites could be assayed in a single injections. This combinatorial approach (Fig 8) can also be used for testing clinical utility of biomarkers in clinical samples as well as to compare different conditions when performing *in vitro* or *in vivo* experiments. Furthermore, we present the utility of using MS in order to reveal the differences in ELV metabolomic profiles of EMT-induced PANC1cell line (as compared to control) and EC samples (as compared to controls). We further propose that the low abundance biomarkers discovered using ELVs from bodily fluids could then be analyzed using targeted mass spectrometry from plasma directly since the sensitivity would be greatly enhanced. The approach described here can be generically used for biomarker identification of cancer or other pathologies thus furthering the precision medicine paradigm. Thus findings from our study have great value given that not only do these present a standardized approach for mass spectrometry based metabolomics profiling, but also provide evidence for using ELVs metabolomics (derived from human fluids as well as from cell culture media) as a rich matrix for biomarker discovery for studies with basic science, clinical or translational focus.

## Conclusions

Biomarker detection and characterization from complex biological matrices, especially for markers that are low in abundance, remains a challenge. There is increasing evidence that underscores the importance of ELVs as carriers of important cellular information that can be used for defining specific changes in health and disease. Herein, we present an experimental pipeline that can be broadly applied for identification of low abundance biomarkers and characterization in clinical samples. Finally, we demonstrate that ELVs represent an untapped source for metabolic and lipidomic biomarker discovery with high clinical and translational relevance. We believe that the methodology presented here will provide an impetus to the growing field of metabolomics underscoring its ability to delineate biomarkers that can be used for pre-clinical detection as well as for following the natural history of disease progression.

## Supporting Information

S1 Fig Analysis of protein expression by western blot of Exosome-Like Vesicles (ELVs) and microvesicles (MVs) isolated from human plasma (2000, 1000 and 500 μL). Expression was also analyzed in non-purified total plasma (T Plasma). Markers CD81, TSG101, Rab5 and Flotillin 1 were blotted in the same membrane (Panel A) and CD63 and CD9 were blotted in an independent membrane (Panel B). Expression of the soluble protein haptoglobin was analyzed in MVs and ELVs isolated from different volumes of T Plasma.
(TIF)

S1 Table Patient clinic-pathological information. (XLSX)

S2 Table Coefficient of variation of Internal Standards in each group of samples. (XLSX)

S3 Table Comparative analyses of putative features identified in human plasma ELVs. Ratios of relative intensities for a selection of metabolites is represented.
(XLSX)

S4 Table Metabolome profiling of ELVs isolated from human plasma samples and cell culture media. **Table A**. Putative identifications in plasma ELVs. **Table B**. Putative identifications in PANC 1 cell culture media ELVs. The metabolite ID, mass to charge ratio (m/z), mass error (ppm), retention time (min) and electrospray ionization mode are detailed.
(XLSX)

S5 Table Unassigned metabolites IDs found in plasma and cell culture media ELVs. (XLSX)

S6 Table Significant m/z detected in ELVs isolated from PANC1 cell culture media. Mass to charge ratio (m/z), fold change (FC), p-value (p-v) and electrospray ionization mode are detailed.
(XLSX)
