# Supplementary figures and images for: Enabling Metabolomics Based Biomarker Discovery Studies Using Molecular Phenotyping of Exosome-Like Vesicles
Source: PLoS One. 2016 Mar 14;11(3):e0151339. doi: 10.1371/journal.pone.0151339 (PMC4790956; doi:10.1371/journal.pone.0151339)

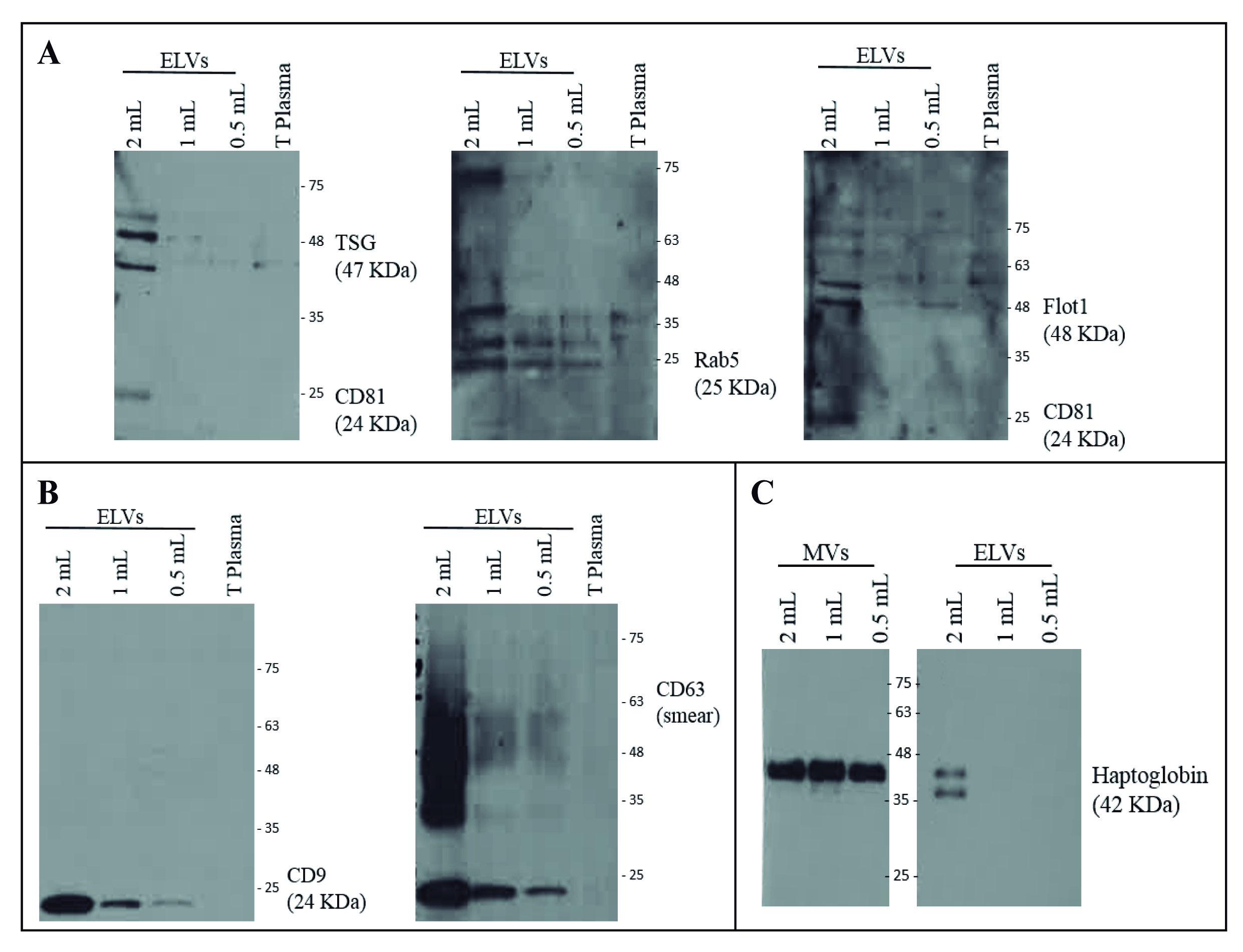

Supplement: S1 Fig — Expression was also analyzed in non-purified total plasma (T Plasma). Markers CD81, TSG101, Rab5 and Flotillin 1 were blotted in the same membrane (Panel A) and CD63 and CD9 were blotted in an independent membrane (Panel B). Expression of the soluble protein haptoglobin was analyzed in MVs and ELVs isolated from different volumes of T Plasma. (TIF) [file pone.0151339.s001.tif]
